# Ultradeep targeted sequencing reveals low allele frequencies of somatic JAK2 and MPL variants in patients with abdominal vein thromboses: results of an ongoing prospective prevalence study in Mecklenburg-West Pomerania

**DOI:** 10.1186/s40364-020-00254-9

**Published:** 2020-12-14

**Authors:** Luise Grunwald, Christina Grosse-Thie, Sina Sender, Gudrun Knuebel, Saskia Krohn, Catrin Roolf, Christian Junghanss, Larissa Henze, Hugo Murua Escobar

**Affiliations:** Department of Medicine, Clinic III - Hematology, Oncology, Palliative Medicine, Rostock University Medical Center, University of Rostock, Ernst-Heydemann-Str. 6, D-18055 Rostock, Germany

**Keywords:** Molecular genetics, Blood coagulation, Myeloproliferative neoplasms, Splanchnic vein thrombosis, Next generation sequencing, Ultradeep targeted sequencing, Low variant allele frequencies, JAK2, MPL

## Abstract

Myeloproliferative neoplasms are characterized by mutations in JAK2, MPL and CALR genes. Commonly in diagnostics and previous studies mainly sequencing and common PCR techniques under conventional detection limits are used.

Splanchnic vein thromboses are rare, but often appear associated with myeloproliferative neoplasms and represent serious complications.

Herein, blood from patients with abdominal vein thromboses in Mecklenburg-West Pomerania (federal district of northern Germany), included in an ongoing prospective prevalence study, was analyzed by next generation sequencing representing the complete protein coding regions of JAK2, MPL and CALR genes with a coverage of > 2000 reads, therefore an ultradeep targeting approach.

JAK2 V617F mutations were detected in 11/44 patients. In four of these cases allele frequencies ranged below the conventional cut off of 2%. MPL W515R was detected in 3/44 cases in low frequencies.

Very low allele frequencies of JAK2 and MPL variants in patients with abdominal vein thromboses may indicate early manifestations of myeloproliferative neoplasms.

**Supplementary Information:**

The online version contains supplementary material available at 10.1186/s40364-020-00254-9.

To the editor:

Splanchnic vein thromboses (SVT) are discussed as first manifestations of myeloproliferative neoplasms (MPN) [1]. JAK2 V617F single nucleotide variants [2] were described to be found in 25 to 41% of patients with SVT [3]. In addition to JAK2 mutations, MPL and CALR mutations are known as driver mutations for MPN. Conventional qPCR and digital droplet PCR (ddPCR) allow sensitive detection of low variant allele frequencies (VAF), but do not allow the simultaneous complex screening of genetic variants. Further in contrast to next generation sequencing (NGS) an efficient identification of novel somatic variants is not possible. Accordingly, prevalence data of JAK2, MPL and CALR variants were predominantly collected qualitatively and infrequently considering the actual allele frequency [4].

Ultradeep NGS combines the advantages of high sensitivity and of simultaneously analyzing multiple gene loci. We analyzed the prevalence of JAK2, MPL and CALR variants affecting the respective protein coding sequences using an ultradeep targeted sequencing approach.

A prospective prevalence study was started in Mecklenburg-West Pomerania to elucidate the prevalence of JAK2 mutations in patients with abdominal vein thromboses. The study included patients from Mecklenburg-Western Pomerania who suffered from current or previous abdominal vein thromboses. The patient’s written consent and a completed clinical questionnaire was required to analyze the blood sample within the study. There were no exclusion criteria. In blood samples from 44 patients with abdominal vein thromboses we detected in thirteen cases JAK2 V617F and MPL W515R variants including seven mutations below the conventional NGS detection limit of 2%: four JAK2 V617F variants as well as three MPL W515R variants (Table 1). One case showed the co-presence of the JAK2 V617F mutation with MPL W515R (Table 1). CALR type I and II mutations were not detected. Besides the hot spot mutations 170 further mutations were detected (data not shown).

**Table 1:** Thrombus localization in patients affected by *JAK2 V617F* and/or *MPL W515R* mutations (n.k. = not known)

Sample No.	JAK2 p.V617F; c.1849G > T; NM_004972.3		MPL p.W515R; c.1543 T > C; NM_005373.2		Portal vein	Hepatic veins	Splenic vein	Mesenteric veins	Other veins	Which
	VAF	Base coverage	VAF	Base coverage						
MPN11	13.2%	7521			+	n.k.	+	+	+	Confluens venae portae
MPN18	31.4%	35,994			+	n.k.	n.k.	n.k.	n.k.	
MPN30	24.6%	11,120			+	n.k.	n.k.	n.k.	n.k.	
MPN38	43.0%	3762			+	n.k.	n.k.	n.k.	n.k.	
MPN42	16.8%	7886			+	+	+	n.k.	–	
MPN44	13.2%	11,159			+	–	–	–	–	
MPN43	28.2%	33,106	0.2%	19,432	+	n.k.	n.k.	n.k.	n.k.	
MPN33			1.2%	28,368	–	–	–	+	–	
MPN46			0.4%	8303	+	n.k.	n.k.	n.k.	n.k.	
MPN28	0.2%	5962			+	n.k.	n.k.	n.k.	n.k.	
MPN34	0.6%	8567			+	–	+	+	n.k.	
MPN36	1.4%	10,976			–	–	–	–	+	Vena renalis sinistra
MPN45	0.3%	7628			+	–	–	–	+	Plexus venosus uteri

Clinical data of the thirteen patients affected by JAK2 and MPL hot spot variants showed a variety in thrombus localization, with portal vein thromboses being the most common (11/13) (Table 1). In three patients with VAF > 2% for the JAK2 V617F mutation splenomegaly was present. Blood cell counts were distributed over a wide range with no obvious differences between patients with VAF > 2% and <  2% (Fig. 1). We noticed a tendency for patients with VAF <  2% to be older (mean age 66.5 years, range 60 to 77 years) compared to patients carrying mutations in higher allele frequencies (mean age 61.5 years, range 48 to 73 years). This is possibly linked to age-related clonal hematopoiesis (ARCH) where the JAK2 V617F mutation has been observed recently [5].

**Fig. 1:**
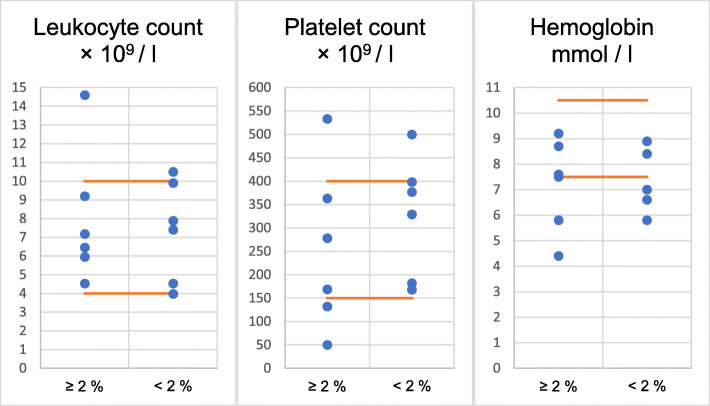
Blood cell counts of patients with mutation of *JAK2 V617F* or *MPL W515R* according to variant allele frequencies: Frequency above the conventional detection limit (> 2%) versus low allele frequency (< 2%). Orange lines show the minimum and maximum limits of the standard values

Most previous studies in patients with SVT reported the prevalence of JAK2 V617F mutation, seldom informing about the sensitivity of the applied method [6] and rarely reporting allele frequencies [4]. By ultradeep targeted sequencing here seven additional mutations were observed, therefore increasing the prevalence of MPN-related mutations in our cohort of 44 patients from 15.9 to 29.5%. These findings support the currently discussed assumption that SVT may represent early manifestations or even precursors of MPN [1]. This is in line with a cohort study of 152 patients with SVT by Colaizzo et al. [6]: During a median follow-up time of 64 months nine patients with presence of JAK2 V617F at the time of SVT developed MPN and six new cases of JAK2 V617F mutated MPN were detected.

The existence of low and ultralow cancer associated VAF is of interest as the lately identified ARCH may represent a pre-manifestation event in patients showing no further criteria for hematologic neoplasms [7]. Follow-up data and studies on larger patient cohorts are warranted to further illustrate the relation of JAK2 and MPL ultralow VAF in the pathogenesis of SVT per se and the manifestation of MPN.

In view of highly sensitive molecular diagnostic tools conventional diagnostic criteria and definition of pathological conditions/diseases may need reconsideration.

## Supplementary Information

**Additional file 1.**

## Data Availability

The datasets used and/or analysed during the current study are available from the corresponding author on reasonable request.

## Abbreviations

ARCH: age-related clonal hematopoiesis
CALR: Calreticulin receptor
ddPCR: digital droplet polymerase chain reaction
DNA: Deoxyribonuclease Acid
JAK2: Janus kinase 2
l: Liter
mmol: Millimole
MPL: Thrombopoietin receptor
MPN: Myeloproliferative neoplasms
ng: Nanogram
NGS: Next Generation Sequencing
n.k.: Not known
No.: Number
PCR: Polymerase chain reaction
SVT: Splanchnic vein thromboses
VAF: Variant allele frequencies
WHO: World Health Organization
