# Supplementary material for: Ultradeep targeted sequencing reveals low allele frequencies of somatic JAK2 and MPL variants in patients with abdominal vein thromboses: results of an ongoing prospective prevalence study in Mecklenburg-West Pomerania
Source: Biomark Res. 2020 Dec 14;8:73. doi: 10.1186/s40364-020-00254-9 (PMC7737343; doi:10.1186/s40364-020-00254-9)
Supplement: Supplementary file 1 — Additional file 1. [file 40364_2020_254_MOESM1_ESM.docx]

**Supplements**

Methods - NGS

Genomic DNA was isolated from peripheral blood cells (NucleoSpin Tissue, Macherey&Nagel). Quantification was done using Qubit 2.0 fluorometer system (Thermo Fisher Scientific). Targeted sequencing library construction was performed using a custom designed Ion AmpliSeq™ NGS Panel (Thermo Fisher Scientific). The panel covers protein coding sequences of the JAK2, MPL, and CALR genes using 82 amplicons. Hot spot regions were covered by at least two amplicons. Sensitivity of the panel was evaluated using genomic DNA carrying JAK2, MPL and CALR hot spot mutations in low VAF.

10 ng DNA per sample was used to amplify, according to the reference sequences annotated in human genome assembly hg19. Sequencing was performed on an Ion Torrent™ Personal Genome Machine™ System. Variant calling and allele frequency determination was performed using Torrent Suite™ software, the variant caller plugin version 5.10.0.18 (Thermo Fisher Scientific) and the Integrated Genome Viewer Version 5.01.

Methods - prevalence trial

Blood samples were collected from patients with abdominal vein thromboses in Mecklenburg-West Pomerania (a federal state of northern Germany), included in an ongoing prospective prevalence study (“Prevalence of JAK2 Mutations in Patients with Abdominal Vein Thromboses”, ethics approval by the ethic committee of Rostock University Medical Center). In total, 44 patients were included between February 2017 and April 2019.
